# Parenting practices and oral health behaviors of children in rural Egypt: gender differences in a household survey

**DOI:** 10.1186/s12903-022-02054-z

**Published:** 2022-01-26

**Authors:** Maha El Tantawi, Nourhan M. Aly, Sara Atteya, Enas Abdellatif, Randa Yassin

**Affiliations:** Pediatric Dentistry and Dental Public Health Department, Faculty of Dentistry, Alexandria University, Champolion St., Azarita, Alexandria, 21527 Egypt

**Keywords:** Parenting, Rural population, Oral health, Toothbrushing, Sugar consumption, Egypt

## Abstract

**Background:**

Parenting practices influence children’s health and development. The current study aimed at assessing gender differences in children’s perception of the parenting practices of both parents, and in the association between children’s oral health behaviors and parenting practices.

**Methods:**

A cross-sectional household survey of 6–12 year old children was conducted in a rural area in Northwestern Egypt in 2019. Clinical examination assessed caries and gingivitis. The Alabama Parenting Questionnaire assessed parenting practices in five domains (positive parenting, involvement, inconsistent disciplining, poor monitoring and corporal punishment) and the World Health Organization questionnaire assessed oral health behaviors including sugar consumption and daily toothbrushing. Sugar consumption was the dependent variable in linear regression and daily toothbrushing was the dependent variable in logistic regression. Parenting practices were the explanatory variables adjusting for confounders. Effect modification by child gender was assessed.

**Results:**

The response rate was 94.1% (n = 433), mean age = 9.9 years, 44.1% boys, 17.8% with daily toothbrushing and mean sugar consumption score = 3.4/8. Girls perceived more mothers’ positive parenting than boys (mean = 14.15 and 13.46) and boys perceived more poor monitoring and corporal punishment. Boys and girls differed in the association between sugar consumption and fathers’ inconsistent disciplining, poor monitoring (*P* = 0.004 and 0.02) and mothers’ corporal punishment (*P* = 0.02), and also daily toothbrushing and mothers’ involvement, positive parenting (*P* = 0.05 and 0.02), fathers’ positive parenting (*P* = 0.02), mothers’ inconsistent discipling and poor monitoring (*P* = 0.01 and 0.04).

**Conclusions:**

There were differences between boys and girls in perceiving mothers’ and fathers’ parenting practices and in the association between these parenting practices and toothbrushing and sugar consumption.

**Supplementary Information:**

The online version contains supplementary material available at 10.1186/s12903-022-02054-z.

## Introduction

Oral health is directly affected by society and culture [1]. Social and cultural factors affect health literacy, access to and demand for care and feeding habits and all these factors are known to affect oral health [2]. Religiosity also differs between cultures and societies and was associated with oral health [3]. Fisher-Owens et al. [4] specified key determinants of children’s oral health including the social environment such as socio-economic status, social support, family function, coping skills of the family, social capital, and culture. The family is the social foundation where children develop behaviors early in life*.* Parents use parenting practices, intentionally or unintentionally to influence children’s health and development [5]. A parenting style composed of favorable and effective parenting practices contributes to healthy child development by influencing dietary habits, physical activity [6], and oral health behaviors [7–10] including the quantity and frequency of sugar intake [5, 11, 12] and oral hygiene habits [13, 14].

The associations between parenting practices and oral health behaviors vary by region and cultural context since parenting practices are affected by the prevailing societal norms. In some settings, parents, especially fathers, are considered the primary authority figures in their children’s lives influencing their developing personalities [15]. Previous studies assessed the association between parenting and oral health behaviors [7–10]. Only few studies are available outside western and southeastern countries [10, 16] and evidence is needed from countries with different cultural backgrounds.

People living in rural settings may be exposed to stressors such as geographic isolation, limited access to healthcare services, and high poverty rates. These additional stresses may affect parenting practices leading to inadequate support for children, negative disciplining, or responding to problems in an abusive or neglectful manner. Also, the extensive social networks in rural communities make parents more likely to seek and receive advice from others living in their community than from healthcare professionals with possible risks for oral health [17]. Most Egyptian children younger than 15 years of age live in rural areas [18] where the poverty rates are higher than in urban areas with less education [19]. Our previous research in rural Egypt showed high sugar consumption and caries prevalence with low frequency of toothbrushing among children and indicated that parenting practices explained an amount of variation in caries experience, plaque accumulation and gingivitis that was similar to oral health behaviors [16]. These factors place Egyptian children living in rural areas at high risk for oral diseases.

Evidence shows differences between mothers’ and fathers’ parenting practices [16] and between parenting practices used with sons and daughters [20]. However, it is not known whether these differences would impact the association between parenting practices and oral health behaviors*.* This information is needed to guide the design of health education programs. This study aimed at assessing gender differences in (1) how children perceive the parenting practices of fathers and mothers and (2) the association between children’s oral health behaviors and parenting practices. The null hypothesis of the study was that there is no difference by child gender in the association between oral health behaviors and mothers’ and fathers’ parenting practices.

## Methods

This was a secondary analysis of data from a study assessing the association between parenting practices and oral health status [16]. The primary study was a cross-sectional household survey of children living in rural areas in Northwestern Delta, Egypt conducted from May 2019 to February 2020. Ethical approval was obtained from the Research Ethics Committee, Faculty of Dentistry, Alexandria University, Egypt (IRB 00010556 – IORG 0008839). Parental consent and children’s assent to participate were secured and the study was conducted in full accordance with the Helsinki declaration.

Multistage random sampling was used: In stage 1, the most populated administrative center in Northwestern Delta was selected [21]. In stage 2, villages were randomly selected. In stage 3, a local guide in each village helped randomly select a household from each street in the village. In the last stage, a cluster sample was used to include all eligible children per household. Children were eligible if they were residents of rural areas, aged between 6 and 12 years, with their mother/female caregiver available in the same household. Intellectually disabled and preschool-age children were excluded because they would not be able to understand the questionnaires. For the original study, children were recruited from four villages based on 95% confidence level to detect caries among Egyptian children of mean (SD) DMFT = 1.04 (1.56), mean (SD) deft = 4.21 (3.21) and reported daily toothbrushing prevalence = 48.09%. [16, 22]. The required number of children was calculated to range from 366 to 384 children. [23] (MedCalc Statistical Software version 18.2.1 (MedCalc Software bvba, Ostend, Belgium; Available from: http://www.medcalc.org; 2018). We also made sure that the present study was adequately powered to detect the smallest effect size in regression analysis based on Newsom’s method (n ≥ 300) [24].

Data were collected from children using interview-based questionnaires and clinical examination. Each child was interviewed separately away from other family members to ensure unbiased responses. The interview included 2 questionnaires; the short form of the child version of the Alabama parenting questionnaire (APQ) which can be used with children aged 6–18 years [25] to assess parenting practices (Additional file 1: Appendix I) and the World Health Organization (WHO) questionnaire-child form assessing oral health behaviors [26]. The APQ measures 5 parenting domains: involvement, supervision and monitoring, use of disciplining techniques, consistency in the use of disciplining and corporal punishment using 15 questions (3 questions per domain). This short version was previously translated into Arabic and validated [27]. All questions were scored on a 5-point Likert scale ranging from 1 (never) to 5 (always). The total score per domain was the sum of scores of the 3 questions. Higher scores on involvement and positive parenting indicate favorable parenting practices, while higher scores on poor monitoring, inconsistent discipline and corporal punishment indicate unfavorable parenting practices. Each child responded twice to the questionnaire to assess maternal and paternal practices. The Arabic version of the WHO questionnaire was used to assess oral health behaviors [13, 26]. This questionnaire assessed the child’s background: sex, age and mother’s education and oral health behaviors: toothbrushing (at least once daily or less), intake of sugary food at least once daily or less including eight types of sugary foods/snacks: fruits, biscuits and cakes, carbonated beverages, jam and honey, sugar-added chewing gums, candies, sugar-sweetened milk and sugar-sweetened hot drinks [13] and dental visits during the previous year (at least once or less). After obtaining parental consent, the questionnaires were pilot tested on 10 children attending the college clinic to ensure appropriateness of the questions and estimate the time needed for response. The questionnaires were further tested in the field on 7 children and their data were excluded from analysis. Data were collected using KoboToolbox, an online platform for offline data collection with subsequent data synchronization when internet access becomes available.

Caries examination was done by three calibrated examiners with Kappa statistic for inter and intra examiner agreement > 0.85. Caries was diagnosed in all teeth at the cavitation level using the WHO criteria [26] under natural daylight using disposable mirrors and ball ended probes #550B. The number of permanent (DMFT) and primary (dft) teeth with caries experience was recorded. Gingivitis was assessed for 6 index teeth (#16/55, #12/52, #24/64, #36/75, #32/72 and #44/84) using the gingival index of Löe and Silness [28].

Sugar consumption score was the sum of points for the eight items consumed daily and ranged from 0 to 8 with higher score indicated greater daily sugar consumption. T-test, chi-squared or Fisher exact tests were used to compare boys and girls regarding background factors, oral health behaviors, clinical oral health parameters and perceived parenting practices. Two types of models were constructed: a linear regression model where the dependent variable was sugar consumption score and a logistic regression model where the dependent variable was daily toothbrushing (yes/no). Explanatory variables in the models were the five parenting practices for mothers and fathers and we adjusted for the confounders which were the sociodemographic factors (child’s age, child’s sex, mother’s education, and village of residence) and history of dental visits. We calculated the *p* values for the interaction or effect modification by child gender. The models were constructed for the whole sample and for boys and girls separately. Adjusted regression coefficients (B), odds ratios (AORs) and 95% confidence intervals (CIs) were calculated. Significance level was set at *p* < 0.05. IBM SPSS for Windows (version 23.0) was used for statistical analysis.

## Results

A total of 433/460 children returned complete questionnaires (response rate = 94.1%). Table 1 shows that there were 191 (44.1%) boys. Boys were significantly younger than girls (mean age = 9.54 and 10.24 years, *P* = 0.02), had higher daily sugar consumption score (mean = 3.49 and 3.32, *P* = 0.33), significantly fewer permanent teeth with caries experience (mean DMFT = 0.48 and 0.70, *P* = 0.04), and significantly more decayed and filled primary teeth (mean dft = 3.38 and 2.73, *P* = 0.03). Lower percentage of boys than girls had mothers with high school or higher education (11.0% and 12.8%, *P* = 0.56), brushed their teeth daily (13.6% and 21.1%, *P* = 0.04) while a higher percentage of boys visited the dentist in the last year (44.0% and 40.9%, *P* = 0.52). There was no significant difference in gingivitis between boys and girls (mean index = 1.15).

**Table 1:** Comparison between sons and daughters regarding background factors, oral health behaviors, clinical parameters and parenting practices in rural Egypt (n = 433)

Factors		Mean (SD)			*P* value
		Overall (n = 433)	Sons (n = 191)	Daughters (n = 242)	
Age in years		9.9 (3.0)	9.5 (2.9)	10.2 (3.1)	0.02*
Mother with high school or higher education	N (%)	52 (12.1)	21 (11.0)	31 (12.8)	0.56
Dental visit last year	N (%)	183 (42.3)	84 (44.0)	99 (40.9)	0.52
Brushing daily	N (%)	77 (17.8)	26 (13.6)	51 (21.1)	0.04*
Sugar consumption score		3.4 (1.8)	3.49 (1.85)	3.32 (1.83)	0.33
DMFT		0.59 (1.13)	0.48 (1.04)	0.70 (1.23)	0.04*
dft		3.06 (3.19)	3.38 (3.29)	2.73 (3.08)	0.03*
Gingival index		1.15 (0.36)	1.15 (0.36)	1.15 (0.38)	0.18
Involvement	Mother	10.60 (3.33)	10.65 (3.45)	10.56 (3.24)	0.78
	Father	8.59 (3.95)	8.99 (4.02)	8.28 (3.86)	0.06
Positive parenting	Mother	13.85 (2.37)	13.46 (2.69)	14.15 (2.03)	0.004*
	Father	12.22 (3.90)	12.45 (3.71)	12.04 (4.04)	0.29
Inconsistent disciplining	Mother	10.45 (3.33)	10.26 (3.59)	10.59 (3.12)	0.32
	Father	8.06 (3.73)	8.06 (3.86)	8.07 (3.63)	0.99
Poor monitoring	Mother	4.45 (2.60)	4.81 (2.51)	4.16 (2.63)	0.01*
	Father	4.31 (2.22)	4.68 (2.30)	4.02 (2.11)	0.002*
Corporal punishment	Mother	8.48 (4.09)	9.17 (4.05)	7.94 (4.06)	0.002*
	Father	7.15 (4.23)	8.98 (4.27)	5.71 (3.60)	< 0.0001*

*Statistically significant at *p* < 0.05

Overall, the children reported greater awareness of mothers’ parenting practices than they did for fathers. There was no significant difference between boys and girls in the perception of mother’s (*P* = 0.78) or father’s involvement (*P* = 0.06), father’s positive parenting (*P* = 0.29), and mother’s (*P* = 0.32) or father’s (P = 0.99) inconsistent disciplining. Boys had significantly lower perception of mother’s positive parenting than girls (mean = 13.46 and 14.15, *P* = 0.004), greater perception of mothers’ (mean = 4.81 and 4.16, P = 0.01) and fathers’ poor monitoring (mean = 4.68 and 4.02, *P* = 0.002) and mothers’ (mean = 9.17 and 7.94, *P* = 0.002) and fathers’ corporal punishment (mean = 8.98 and 5.71, *P* < 0.0001). Mothers had significantly higher involvement, positive parenting and inconsistent disciplining scores than fathers for boys and girls (*P* < 0.05). Also, mothers had significantly higher corporal punishment scores than fathers for girls (*P* = 0.03) whereas mothers’ and fathers’ scores were not significantly different for boys (*P* = 0.41).

Table 2 shows that there were no significant overall associations between sugar consumption and parenting practices. There were no significant differences between boys and girls in the association between sugar consumption and involvement or positive parenting by mothers or fathers (*P* = 0.65, 0.34, 0.33 and 0.73). There were significant differences between boys and girls in the association between sugar consumption and fathers’ inconsistent disciplining (*P* = 0.004) and fathers’ poor monitoring (*P* = 0.02): significantly greater sugar consumption was associated with inconsistent fathers’ disciplining (B = 0.12, 95% CI 0.03, 0.20) and poor monitoring B = 0.20, 95% CI 0.01, 0.40) in boys and less sugar consumption in girls (B = − 0.06, 95% CI − 0.13, 0.01 and B = − 0.15, 95% CI − 0.33, 0.04 respectively). Child gender significantly modified the association between sugar consumption and mother’s corporal punishment (*P* = 0.02): corporal punishment was associated with less sugar consumption in boys (B = − 0.07, 95% CI − 0.15, 0.02) and more sugar consumption in girls (B = 0.05, 95% CI − 0.02, 0.12).

**Table 2:** Association between sugar consumption and parenting practices and effect modification by child’s gender in rural Egypt (n = 433)

Parenting practices	B (95% CI)			*P* for interaction
	Overall	Sons	Daughters	
Mother involvement	0.02 (− 0.06, 0.09)	0.04 (− 0.05, 0.13)	0.02 (− 0.06, 0.09)	0.65
Father involvement	0.03 (− 0.04, 0.09)	0.07 (− 0.01, 0.15)	0.02 (− 0.04, 0.08)	0.34
Mother positive parenting	− 0.04 (− 0.18, 0.08)	0.03 (− 0.08, 0.14)	− 0.02 (− 0.14, 0.09)	0.33
Father positive parenting	0.01 (− 0.06, 0.07)	0.03 (− 0.05, 0.11)	− 0.01 (− 0.07, 0.05)	0.73
Mother inconsistent discipline	0.04 (− 0.05, 0.12)	− 0.02 (− 0.11, 0.07)	0.05 (− 0.03, 0.13)	0.29
Father inconsistent discipline	− 0.05 (− 0.12, 0.03)	0.12 (0.03, 0.20)*	− 0.06 (− 0.13, 0.01)	0.004*
Mother poor monitoring	0.08 (− 0.07, 0.23)	− 0.01 (− 0.19, 0.17)	0.07 (− 0.08, 0.21)	0.36
Father poor monitoring	− 0.12 (− 0.31, 0.07)	0.20 (0.01, 0.40)*	− 0.15 (− 0.33, 0.04)	0.02*
Mother corporal punishment	0.07 (− 0.01, 0.14)	− 0.07 (− 0.15, 0.02)	0.05 (− 0.02, 0.12)	0.02*
Father corporal punishment	− 0.02 (− 0.10, 0.06)	0.10 (0.01, 0.18)*	− 0.02 (− 0.10, 0.05)	0.07

Adjusting for village, age, mother’s education, and dental visits

*B* adjusted regression coefficient, *CI* confidence interval

*Statistically significant at *p* < 0.05

There were significant associations between daily toothbrushing and inconsistent disciplining: mothers’ inconsistent disciplining was associated with higher odds of daily toothbrushing (AOR = 1.22, 95% CI 1.06, 1.41) and fathers’ inconsistent disciplining was associated with lower odds of daily toothbrushing (AOR = 0.85, 95% CI 0.75, 0.96, Table 3). Child gender significantly modified the association between daily toothbrushing and mothers’ involvement (*P* = 0.05), mothers’ positive parenting (*P* = 0.02) and fathers’ positive parenting (*P* = 0.02): mothers’ involvement and positive parenting were associated with lower odds of daily toothbrushing in boys (AOR = 0.80 and 0.60) and higher odds in girls (AOR = 1.01 and 1.05). Father’s positive parenting was associated with higher odds of toothbrushing in boys (AOR = 1.15, 95% CI 0.99, 1.33) and lower odds in girls (AOR = 0.86, 95% CI 0.76, 0.97). There were significant differences between boys and girls in the association between daily toothbrushing and mothers’ inconsistent discipline (*P* = 0.01) and mothers’ poor monitoring (*P* = 0.04). These practices were associated with lower odds of daily toothbrushing in boys (AOR = 0.92, 95%CI 0.79, 1.07 and AOR = 0.66, 95%CI 0.48, 0.93) and higher odds in girls (AOR = 1.24, 95%CI 1.07, 1.44 and AOR = 1.09, 95%CI 0.85, 1.40).

**Table 3:** Association between daily toothbrushing and parenting practices and effect modification by child’s gender in rural Egypt (n = 433)

Parenting practices	AOR (95% CI)			*P* for interaction
	Overall	Sons	Daughters	
Mother involvement	1.03 (0.92, 1.15)	0.80 (0.67, 0.96)*	1.01 (0.90, 1.14)	0.045*
Father involvement	0.93 (0.85, 1.03)	1.10 (0.96, 1.25)	0.92 (0.83, 1.02)	0.13
Mother positive parenting	1.05 (0.86, 1.29)	0.60 (0.40, 0.90)*	1.05 (0.85, 1.31)	0.02*
Father positive parenting	0.89 (0.79, 1.00)	1.15 (0.99, 1.34)	0.86 (0.76, 0.97)*	0.02*
Mother inconsistent discipline	1.22 (1.06, 1.41)*	0.92 (0.79, 1.07)	1.24 (1.07, 1.44)*	0.01*
Father inconsistent discipline	0.85 (0.75, 0.96)*	1.02 (0.89, 1.17)	0.83 (0.73, 0.94)*	0.07
Mother poor monitoring	1.05 (0.82, 1.33)	0.66 (0.48, 0.93)*	1.09 (0.85, 1.40)	0.04*
Father poor monitoring	0.96 (0.72, 1.29)	1.52 (1.001, 2.30)*	0.94 (0.69, 1.27)	0.08
Mother corporal punishment	1.08 (0.97, 1.20)	1.03 (0.89, 1.19)	1.07 (0.95, 1.20)	0.58
Father corporal punishment	1.07 (0.93, 1.22)	1.09 (0.94, 1.27)	1.07 (0.93, 1.23)	0.94

Adjusting for village, age, mother’s education, and dental visits

*AOR* adjusted odds ratio, *CI* confidence interval

*Statistically significant at *p* < 0.05

## Discussion

The study showed that in rural Egypt, girls were more likely to report positive parenting by mothers and boys to report poor monitoring and corporal punishment. Girls received more corporal punishment from mothers than from fathers and both parents gave boys more corporal punishment than girls. There were no significant overall associations between daily sugar consumption and parenting practices. Daily toothbrushing was associated with inconsistent disciplining with differences by parent gender. Gender-specific differences were observed in the association between oral health behaviors and some parenting practices, with direct associations between these practices in similar sex dyads (fathers/boys and mothers/girls) for sugar consumption and toothbrushing. Thus, the null hypothesis of the study is rejected.

The study had several strengths. First, interviewing children instead of parents about parenting practices helped reduce social-desirability bias. Second, identifying maternal and paternal practices separately and splitting responses by child gender allowed the assessment of gender-specific associations and showed associations that would be hidden if combined data were used. Third, we used a household survey which confers greater generalizability than studies recruiting children from pediatric dental clinics or schools. The study, however, had some limitations. Its cross-sectional design does not support causality and cannot verify time sequence and thus, longitudinal studies are needed for confirmation. Also, reporting on sugar consumption and toothbrushing may be liable to recall and social desirability biases. In addition, some of the observed associations or differences may be confounded by factors that were not included in the study and that should be taken into consideration in future studies such as the locus of control of children, the relationship between both parents, family functioning as well as the oral health practices of the parents themselves. The present findings are limited to children of the same age living in rural areas. Research shows that the Egyptian society is generally conservative and religious [29] and these factors were associated with specific parenting practices such as less acceptability of corporal punishment of children [30]. These characteristics are shared by similar communities in other Arab and Middle Eastern countries. Further studies in various settings including populations with different characteristics can identify differences and similarities. Furthermore, the cultural and social profile of the rural setting of the study may differ from urban settings in Egypt and elsewhere.

The children in the present study had greater perception of mothers’ than fathers’ parenting practices possibly reflecting greater presence of mothers than fathers at home. Fathers are more likely to be away from home for work commitments [31] and mothers are mostly involved in household and domestic work, thus spending more time at home than men in rural setting and women in urban settings [32]. The findings also showed that parents in rural areas may be using a parenting style for boys that is reactionary and characterized by poor supervision with physical punishment. Awareness programs need to highlight the consequences of this parenting style and its detrimental effect on child’s development and adoption of proper health behaviors.

The present study did not show that favorable parenting practices were associated with less poor or more favorable oral health behaviors. This disagrees with the literature showing the impact of parenting styles on other health practices. For example, American adolescents reported a protective effect of parental monitoring against using tobacco, marijuana, alcohol, and sex initiation [33]. Arab adolescents had more physical activity with authoritative parenting [34]. Czech adolescents were more involved in excessive internet use in the presence of authoritarian than authoritative parenting styles [35]. On the other hand, studies assessing the relationship between parenting practices and oral health behaviors had contradicting conclusions. A Kenyan study showed that parental monitoring was associated with lower levels of poor oral hygiene [36] and an Indian study showed a negative association between children's oral hygiene behaviors and parental power assertion [9]. On the other hand, a study of Lithuanian children reported no association between toothbrushing and authoritative parenting [37]. These differences may be attributed to cultural characteristics or the inclusion of different health practices. Further research is needed to confirm whether oral health behaviors are affected by parenting styles like other health habits.

The present study showed differences in the association between children’s oral health behaviors and parenting practices by parent and child gender: in similar-sex dyads, children oral health behaviors were directly associated with parenting practices and in opposite-sex dyads, they were inversely associated. Studies combining the practices of both parents and the effect on children regardless of child’s gender may obscure important differences and associations. Thus, our study fills a knowledge gap by focusing on gender differences in these associations. Our findings agree with studies showing differences between the effect of mothers’ and fathers’ parenting styles on boys’ and girls’ health behaviors including use of condoms, early sexual initiation and use of psychoactive substances [38, 39]. However, the present findings do not agree with a study reporting that parenting practices and body mass index were associated in opposite-sex dyads of American adolescents and their parents [40].

Our findings apply to similar rural populations and children of similar ages. Caution is advised in generalizing to populations in urban settings and to adolescents where changes in the balance of power in the family environment may introduce new dimensions to the child-parent relationship. Also, a small percentage of children in the present study brushed their teeth daily and this, in addition to the low level of caries and gingivitis, may need to be taken into consideration when generalizing the findings to populations with greater oral health problems where the impact of parenting practices may be more pronounced. The study showed that parenting practices may affect how children care for their own oral health. Thus, in addition to educating parents about how children should brush their teeth and what they should eat, parents should be advised about the importance of balanced and supportive parenting practices for their children’s wellbeing and oral health. Including both parents in health education programs is important because focusing on mothers only may reduce the chances of educating fathers about the effect of their parenting on boys’ oral health behaviors. However, confirmation is needed from further studies to support solid recommendations that may challenge prevailing societal norms about the gender-specific roles of mothers and fathers in rural areas in bringing up their children.

Further studies are needed to understand the impact that care practices of extended family members may have on the oral health of children in these closely-knit communities. In addition, it is important to assess the relationship between parenting practices and children’s oral health behaviors in single parent families and families with non-traditional structures in similar and other setting.

## Conclusion

Boys and girls differently perceived the parenting practices of mothers and fathers. The association between parenting practices and children’s sugar consumption and daily toothbrushing differed by child and parent gender. Girls had better daily toothbrushing habits and similar sugar consumption to boys with greater perception of positive parenting by mothers and less perception of poor monitoring and corporal punishment by both parents.

## Supplementary Information

**Additional file 1.** Short version of the Alabama Parenting Questionnaire.

## Data Availability

The datasets used and/or analyzed during the current study are available from the corresponding author on reasonable request.

## Abbreviations

APQ: Alabama parenting questionnaire
WHO: World Health Organization
SD: Standard deviation
AOR: Adjusted odds ratio
CI: Confidence interval
