# Supplementary material for: Parenting practices and oral health behaviors of children in rural Egypt: gender differences in a household survey
Source: BMC Oral Health. 2022 Jan 26;22:17. doi: 10.1186/s12903-022-02054-z (PMC8793182; doi:10.1186/s12903-022-02054-z)
Supplement: Supplementary file 1 — Additional file 1. Short version of the Alabama Parenting Questionnaire. [file 12903_2022_2054_MOESM1_ESM.docx]

**Appendix I**

**Short version of the Alabama Parenting Questionnaire**

| Parenting Domain | Item |
| --- | --- |
|  |  |
| Parental Involvement | You play games or do other fun things with your parent |
|  | Your parent asks you about your day in school |
|  | Your parent helps you with your homework |
| Positive parenting | Your parent tells you that you are doing a good job |
|  | Your parent compliments you when you have done something well |
|  | Your parent praises you for behaving well |
| Inconsistent discipline | Your parent lets you out of a punishment early |
|  | Your parent threatens to punish you and then do not do it |
|  | You talk your parent out of punishing you after you have done something wrong |
| Poor supervision and monitoring | You fail to leave a note or let your parent know where you are going |
|  | Your parent let you stay out in the evening past the time you are supposed to be home |
|  | Your parent does not know the friends you are with |
| Corporal punishment | Your parent spanks you with their hand when you have done something wrong |
|  | Your parent hits you with a belt, switch, or other object when you have done something wrong |
|  | Your parent slaps you when you have done something wrong |
